# Impairments of cortico-cortical connectivity in fine tactile sensation after stroke

**DOI:** 10.1186/s12984-021-00821-7

**Published:** 2021-02-15

**Authors:** Sa Zhou, Yanhuan Huang, Jiao Jiao, Junyan Hu, Chihchia Hsing, Zhangqi Lai, Yang Yang, Xiaoling Hu

**Affiliations:** 1 Department of Biomedical Engineering, The Hong Kong Polytechnic University, Hong Kong, China; 2 Institute of Textiles and Clothing, The Hong Kong Polytechnic University, Hong Kong, China

**Keywords:** Stroke, Sensory impairment, Fine tactile sensation, Functional connectivity, Functional brain network

## Abstract

**Background:**

Fine tactile sensation plays an important role in motor relearning after stroke. However, little is known about its dynamics in post-stroke recovery, principally due to a lack of effective evaluation on neural responses to fine tactile stimulation. This study investigated the post-stroke alteration of cortical connectivity and its functional structure in response to fine tactile stimulation via textile fabrics by electroencephalogram (EEG)-derived functional connectivity and graph theory analyses.

**Method:**

Whole brain EEG was recorded from 64 scalp channels in 8 participants with chronic stroke and 8 unimpaired controls before and during the skin of the unilateral forearm contacted with a piece of cotton fabric. Functional connectivity (FC) was then estimated using EEG coherence. The fabric stimulation induced FC (SFC) was analyzed by a cluster-based permutation test for the FC in baseline and fabric stimulation. The functional structure of connectivity alteration in the brain was also investigated by assessing the multiscale topological properties of functional brain networks according to the graph theory.

**Results:**

In the SFC distribution, an altered hemispheric lateralization (HL) (HL degree, 14%) was observed when stimulating the affected forearm in the stroke group, compared to stimulation of the unaffected forearm of the stroke group (HL degree, 53%) and those of the control group (HL degrees, 92% for the left and 69% for the dominant right limb). The involvement of additional brain regions, i.e., the distributed attention networks, was also observed when stimulating either limb of the stroke group compared with those of the control. Significantly increased (P < 0.05) global and local efficiencies were found when stimulating the affected forearm compared to the unaffected forearm. A significantly increased (P < 0.05) degree of inter-hemisphere FC (interdegree) mainly within ipsilesional somatosensory region and a significantly diminished degree of intra-hemisphere FC (intradegree) (P < 0.05) in ipsilesional primary somatosensory region were observed when stimulating the affected forearm, compared with the unaffected forearm.

**Conclusions:**

The alteration of cortical connectivity in fine tactile sensation post-stroke was characterized by the compensation from the contralesional hemisphere and distributed attention networks related to involuntary attention. The interhemispheric connectivity could implement the compensation from the contralateral hemisphere to the ipsilesional somatosensory region. Stroke participants also exerted increased cortical activities in fine tactile sensation.

## Background

Fine tactile sensation plays an important role in motor relearning after stroke, and participates not only in initiating effective motor behaviors but also in fine-tuning subsequent movements for fine motor control [1–3]. It is notable however, that there is a relatively marginal amount of knowledge regarding its dynamics in the process of post-stroke rehabilitation. This is mainly due to a lack of effective evaluation on neural responses to fine tactile stimulation. On the one hand, the traditional measures of fine tactile impairments in clinical practice are disadvantageous in terms of reliability and repeatability without direct cortical detection [3]. For example, the two-point discrimination test depends not only on the pressure applied to the finger by the examiner to induce tactile stimulation, but also on the cognitive and discriminative levels of patients in terms of subtle differences due to the inherently subjective nature of tactile sensation [3]. On the other hand, functional neuroplasticity widely occurs in multiple brain regions, including local and remote areas with respect to the lesion site reorganized after a stroke. This would further result in the cortical reorganization and connectivity disturbance, as previously reported in studies on motor functions [4, 5]. A redistributed pattern from the ipsilesional hemisphere to the contralesional hemisphere is commonly observed during motor or cognitive tasks in stroke participants [6, 7]. However, compared to the extensively studied motor impairments, little is known about the neuroplasticity associated with sensory impairments post-stroke. This is principally due to a lack of evidence regarding the strategies of cortical recruitment particularly in the area of fine tactile sensation.

There are some studies on resting-state functional magnetic resonance imaging (rsfMRI) which examined the changes of cortical recruitment in relation to the tactile impairment post-stroke, as revealed by functional connectivity (FC) [8, 9]. For example, Bannister et al. exploited rsfMRI to examine the relationship between the recovery of tactile sensation and the resting-state FC following a stroke. The results indicated that the changes of resting-state FC between somatosensory regions and distributed regions, including vision and attention networks, were associated with improved tactile sensation within the first 6 months post-stroke [8]. Goodin et al. also used rsfMRI to investigate the effect of different lesion sites in the hemispheres on the functional connectivity of tactile sensation in stroke participants [9]. It was found that the patients with lesions in the right hemisphere had greater intra-hemispheric connectivity from the ipsilesional primary somatosensory cortex (S1) to inferior parietal regions than those with left lesions and unimpaired controls. However, these studies revealed only the alterations of static cortical networks during the resting-state after stroke. Furthermore, fMRI is limited in terms of temporal resolution, despite the advantages of higher spatial resolution and deeper imaging of brain activities beyond the cortical level than electroencephalogram (EEG). In this sense, the fMRI is inadequate when seeking the detection of the cortical activities in transient tactile stimulation, since the sensory neurons change their levels of sensitivity to a constant stimulus over time, i.e., sensory adaptation [10]. Thus, the available results on tactile impairments post-stroke might not be suitable to reveal the strategies of the alteration in cortical connectivity during the tactile sensation, which is a typically transient process [11].

In comparison to fMRI, when evaluating the cortical connectivity, EEG offers a higher degree of temporal resolution when seeking to capture neural activities during transient tasks [12, 13]. In this regard, the EEG-derived FC [14, 15], demonstrating the interaction of information among cortical regions, has been proven to be effective in capturing the alteration of cortical connectivity in transient motor tasks in stroke survivors [16, 17]. For instance, Strens et al. compared the EEG-derived FC during a 25% maximal handgrip task in chronic stroke participants and unimpaired persons [16]. The results revealed greater FC between the ipsilesional supplementary motor area (SMA) and sensorimotor area in the stroke than the unimpaired controls, which might have a dynamically compensatory effect for brain lesion after a stroke. The EEG-derived FC has also been applied to measure the post-stroke alteration in cortical connectivity during the repeated finger extensions with a frequency of 1 Hz. It was found that the intensity of FC between contralesional motor/premotor cortex and SMA was increased in stroke subjects compared with the unimpaired controls [17]. Despite the successful evaluations of the EEG-derived FC for motor neuroplasticity following stroke, its investigation on sensory neuroplasticity has not been well carried out. Such an investigation would have the potential to further develop current understandings of the alteration in cortical connectivity in relation to the tactile impairments post-stroke.

The alteration of cortical connectivity in its functional structure can be visualized by the graph theory-based approach from a network perspective [18, 19], where the EEG channels at different cortical locations and their FCs are topographically represented as nodes and links among them [20]. The graph theory analysis has been adopted to reveal the stroke-induced changes in functional brain networks from local (e.g., single-node connectivity) to global level (e.g., connectivity of the entire brain) represented by indices at difference scales [21]. Thus the examination of the dynamic information processing and neural communication during motor or cognitive tasks was facilitated [22]. De Vico Fallani et al. also examined the functional brain organization in stroke subjects whilst engaging in the finger tapping, where inefficient brain networks were found in stroke participants with a lower capacity to integrate the information from remote brain regions and a lower capacity of processing information in local brain regions, compared with unimpaired persons [21]. Additionally, in a study by Philips et al. on persons with chronic stroke [23], the reduction of graph theoretical indices represented by the parameters of global efficiency, local efficiency and the density of intrahemispheric FC on the unaffected hemisphere was found to correlate with post-stroke motor improvements measured by the increments in the upper-extremity portion of the Fugl-Meyer Assessment (FMUE) after a physical treatment for 12 weeks. However, little has been done using the graph theoretical analysis to understand the functional structure in relation to the connectivity alteration in the brain following the post-stroke tactile impairments.

The purpose of this study was to investigate the post-stroke alteration of cortical connectivity in response to fine tactile stimulation via the textile fabric by EEG-derived functional connectivity analysis. Whole brain EEG was recorded from 64 scalp channels in 8 persons with chronic stroke and 8 age-matched unimpaired controls before and during the unilateral forearm skin contact with cotton fabric. Functional connectivity was then estimated using the EEG coherence method [24]. The fabric stimulation induced functional connectivity (SFC) was analyzed by means of a cluster-based permutation test based on the estimated FC [25]. Furthermore, the multiscale topological properties of functional brain networks were assessed using the graph theory-based method to reveal the functional structure of the connectivity alteration in the brain during transient fine tactile sensation after stroke on multiple levels. Finally, the alteration of brain connectivity in relation to the tactile impairments post-stroke was discussed in detail.

## Methods

### Subject recruitment

This study was approved by the Human Subjects Ethics Subcommittee of Hong Kong Polytechnic University. Before engaging in the study, all participants were informed of the purpose of the research and provided their written consents. We screened the stroke subjects to ensure that they satisfied the following inclusion criteria: (1) More than six months after the onset of unilateral brain lesion due to subcortical stroke; (2) No visual, cognitive or attention deficits as assessed by the Mini-Mental State Examination (MMSE) score > 21 [26]; (3) The spasticity at the wrist and elbow joints was less than 3 as assessed by the Modified Ashworth Scale (MAS) [27]; (4) No neurological impairments except stroke; (5) Moderate to severe sensory impairments on the affected forearm with a score of 1 as measured by the sensation part of light touch in the Fugl-Meyer Assessment (FMA). For the unimpaired subjects, the inclusion criteria were no history of any somatosensory impairments and neurological or psychiatric disorders. Finally, eight stroke subjects were recruited and eight age-matched ($p= 0.56$, independent t-test) unimpaired subjects were recruited as the control group (demographic data and clinical scores were shown in Tables 1 and 2, respectively). All unimpaired participants were right-handed. Given the fact that all stroke participants recruited were at a very chronic stage with at least 10 years after a stroke, their affected and unaffected limbs could be considered as non-dominant and dominant sides, respectively, due to the considerable nonuse of the affected limb and the increased use of the unaffected limb [28].

**Table 1:** Demographic data of the recruited subjects

Group	No. of participants	Stroke types, hemorrhage/ischemic	Affected side, left/right	Gender, male/female	Age (years, mean ± std)	Min/Max years after stroke
Stroke	8	2/6	4/4	7/1	60.8 ± 7.2	10/28
Control	8	–/–	–/–	3/5	57.8 ± 12.1	–/–

**Table 2:** Impairments Measured by Clinical scores in the recruited stroke subjects

Clinical assessment	FMA full motor score	FMA shoulder/ elbow		FMA wrist/ hand	FMA sensory	ARAT	FIM	MAS elbow	MAS wrist	MAS finger
Score (mean ± std)	44.38 ± 16.70	29.55 ± 9.60		14.95 ± 7.28	1	29.28 ± 18.35	65.43 ± 1.98	1.12 ± 0.56	0.87 ± 0.64	0.79 ± 0.51

*FMA *Fugl-Meyer Assessment [65], *FIM *Functional Independence Measurement [66], *MAS * Modified Ashworth Score [27], *ARAT * Action Research Arm Test [67]

### EEG recording during fine tactile stimulation by cotton fabrics

**Fig. 1:**
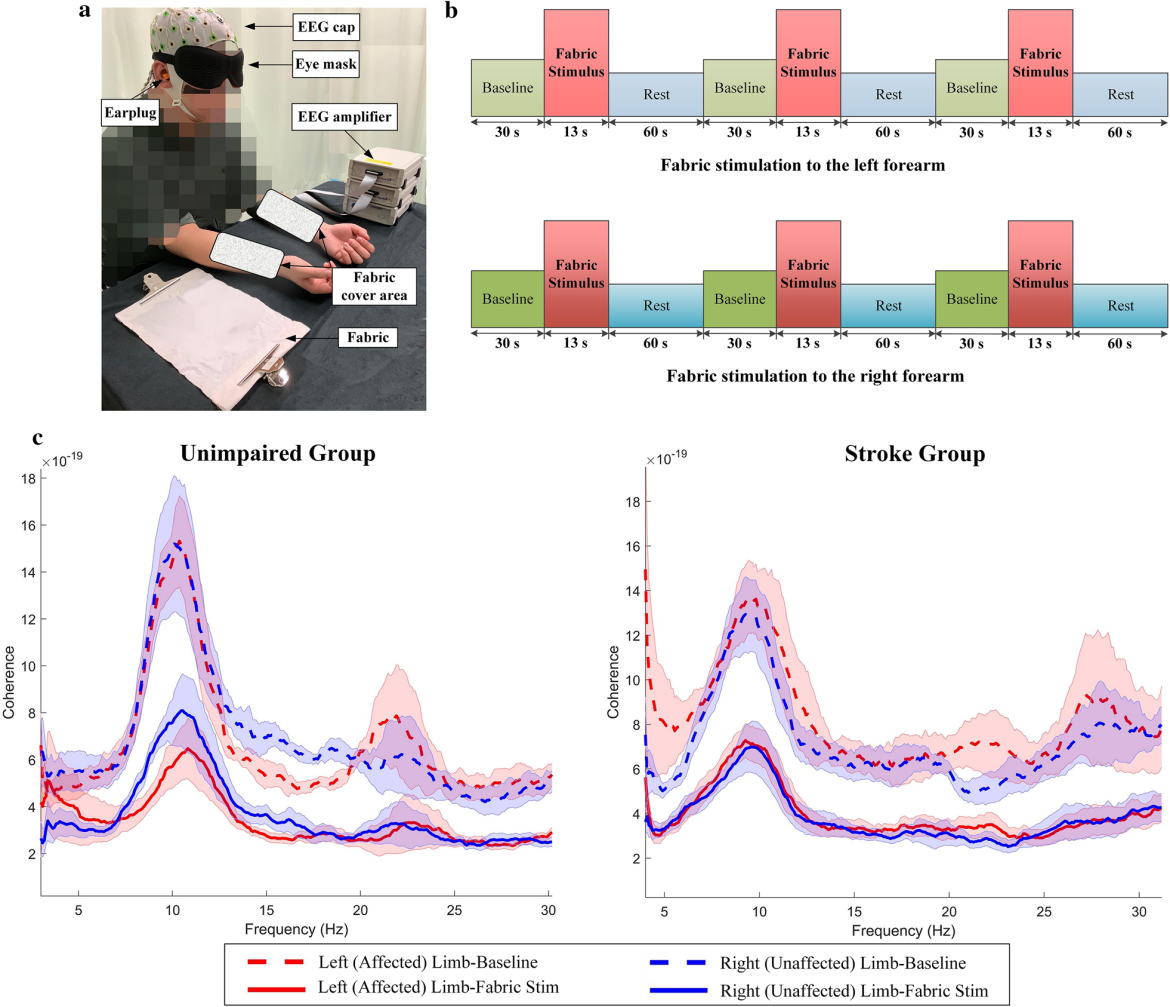
**a** Experimental setup; **b** Experimental protocol presented with timeline; **c** Averaged ImCoh spectra across all channel-pairs and all subjects in unimpaired and stroke groups in each experimental condition (i.e., the baseline and fabric stimulation). The line and shaded area indicate the mean values and the standard error, respectively

The experiment was conducted in a quiet room with the temperature and relative humidity maintained at 18–20 ^◦^C and 60% ± 5%, respectively. Each participant was invited to sit comfortably in front of a table and was instructed to rest the upper limbs on a cushion. Figure 1a shows the experimental setup. Visual and audio interferences to the subject were further minimized by wearing an eye mask and ear plugs. Then, the cap of a 64-channel EEG system for whole brain recording (BP-01830, Brain Products Inc.) was mounted onto the scalp of the participant according to the standard international 10–20 system. Impedance of each electrode was prepared below 5 kΩ. The subject was asked to relax, to close the eyes, not to fall asleep and to avoid any motion or active mental tasks during the EEG recording.

In this work, the fine tactile stimulation was induced by a piece of fabric (100% cotton, plain woven, weight: 127.7 ± 0.8 g/m^2^ thickness: 0.39 ± 0.01 mm, size: 40 × 20 cm) in direct contact with the forearm skin [29]. The textile fabric was deemed to be suited to the experiment as it is a relatively light material and its surface texture is able to evoke sensory responses in corresponding cortical regions [30]. Pure cotton fabric was selected because it is the most commonly used textile fabric in daily life and is generally considered to be comfortable [31]. The fabric would be statically loaded onto the skin of the ventral side of the forearm during stimulation because this area is rich of tactile afferents which are closely related to fine tactile sensation and sensorimotor cortical activation [29, 32].

Figure 1b shows the stimulation protocol presented with the timeline. For each subject, the same stimulation procedure with continuous EEG recordings was performed on the right and left forearms for 3 trials, respectively, in a randomized order. Each single trial contained a baseline test (30 s each) and a fabric stimulus (13 s each). The participant simply relaxed for 60 s following each stimulus. In the baseline test, the subject sat quietly without stimulation throughout the 30 s duration. In each fabric stimulus, the fabric sample was slightly loaded without striking onto the skin surface of the inner forearm and maintaining for 13 s to achieve the fine tactile stimulation. The same experimental protocol was performed for the two groups.

During the experiment, EEG signals were simultaneously recorded with a sampling frequency of 1000 Hz. After the recordings, the EEG signals were preprocessed offline by a band-pass filter from 1 to 45 Hz, re-referenced according to the average of all electrodes and further depurated using the independent component analysis (ICA) to remove artifacts related to possible ocular movements [33]. A visual inspection was also undertaken to reject any segments affected by artifacts. The artifact-free data recorded at the first 12 s starting from the onset of each experimental condition (fabric stimulation or baseline), during which the transient sensory adaptation mainly occurred [34], were retained to ensure the stability of the signals and a consistent data length. Then, EEG signals (62-channels each, the ground and reference were removed) were further segmented into trials of 4 s. In total, 288 trials ($N={N}_{\mathrm{experimental\,condition}}\times {N}_{trial}\times {N}_{forearm}\times {N}_{subject}$, where${N}_{\mathrm{experimental\,condition}}=2$, i.e., fabric stimulation and baseline,${N}_{trial}=9$,${N}_{forearm}=2$, ${N}_{subject}=8$) were generated for each subject group. Finally, the scalp electrode positions of the EEG data were flipped along the mid-sagittal plane for patients with lesions in the left hemisphere to right hemisphere, i.e., the affected hemisphere (Ahemi) and the unaffected hemisphere (Uhemi), respectively, thus allowing for performing a group analysis on all 8 stroke participants and increasing the statistical power as practiced previously in stroke persons [21, 35]. All EEG data processing mentioned above was conducted using the Fieldtrip software (http://www.fieldtriptoolbox.org) supplemented by in-house written code.

### Functional connectivity estimation

Functional connectivity demonstrates the information interaction among different cortical areas. It was estimated by the imaginary coherence (ImCoh) [24] among different EEG channels of the whole brain for the baseline and fabric stimulation trials, as calculated in the motor functional studies previously [21]. The ImCoh can suppress the false connectivity arising from volume conduction (i.e., common mode noise), as the zero-time lag between two EEG signals with the common mode noise can make its ImCoh to be zero [24]. This method yielded weighted values from 0 to 1 for each frequency bin, i.e., the higher the ImCoh in a frequency (e.g., beta band), the stronger the functional connectivity between the cortical regions at that frequency. Therefore, the strength of FC in frequency domain was directly reflected by the ImCoh values. ImCoh is defined as the imaginary part of coherency ${C}_{ij}$, which is the normalized cross-spectrum between the two time series, i.e., the time series of two EEG channels $i$ and $j$:

1 $${C}_{ij}=\frac{{S}_{ij}(f)}{({S}_{ii}(f){S}_{jj}(f){)}^{1/2}}$$

where ${S}_{ij}(f)=<{x}_{i}(f){x}_{j}^{*}(f)>$ is the cross-spectrum between the two time series, * indicates the complex conjugate, $< >$ is the expectation value, ${x}_{i}(f)$ and ${x}_{j}(f)$ are the complex Fourier transforms of $\hat{x}_{i}(f)$ and $\hat{x}_{j}(f)$, respectively.

In each calculation, the EEG time series was partitioned into overlapping (50%) segments with a window length of 2 s, resulting in a frequency resolution of 0.5 Hz. Since there were $n=62$ EEG channels available, the resulting matrix $C$ was antisymmetric with $n(n-1)/2 = 1891$ possible ImCoh values for the whole brain. The FCs in baseline trials were subsequently used for the extraction of fabric stimulation induced FCs from the FCs in fabric stimulation trials.

Beta band (15–30 Hz) was adopted for FC estimation, which has been identified as the key EEG frequency band related with tactile sensation [29, 36]. Figure 1c shows the representative amplitude of ImCoh spectra by mean and std. The amplitude of ImCoh was reduced during the fabric stimulation in the two groups. The peak ImCoh in beta band was statistically different from the baseline when stimulating either limb of the stroke and control groups (P < 0.05, paired t-test). According to the waveform of ImCoh spectra, the beta band was divided into 3 sub-bands, i.e., the beta 1 (15–19 Hz), beta 2 (20–25 Hz) and beta 3 (26–30 Hz), which would be applied to subsequent analyses.

### Functional connectivity induced by fabric stimulation

**Fig. 2:**
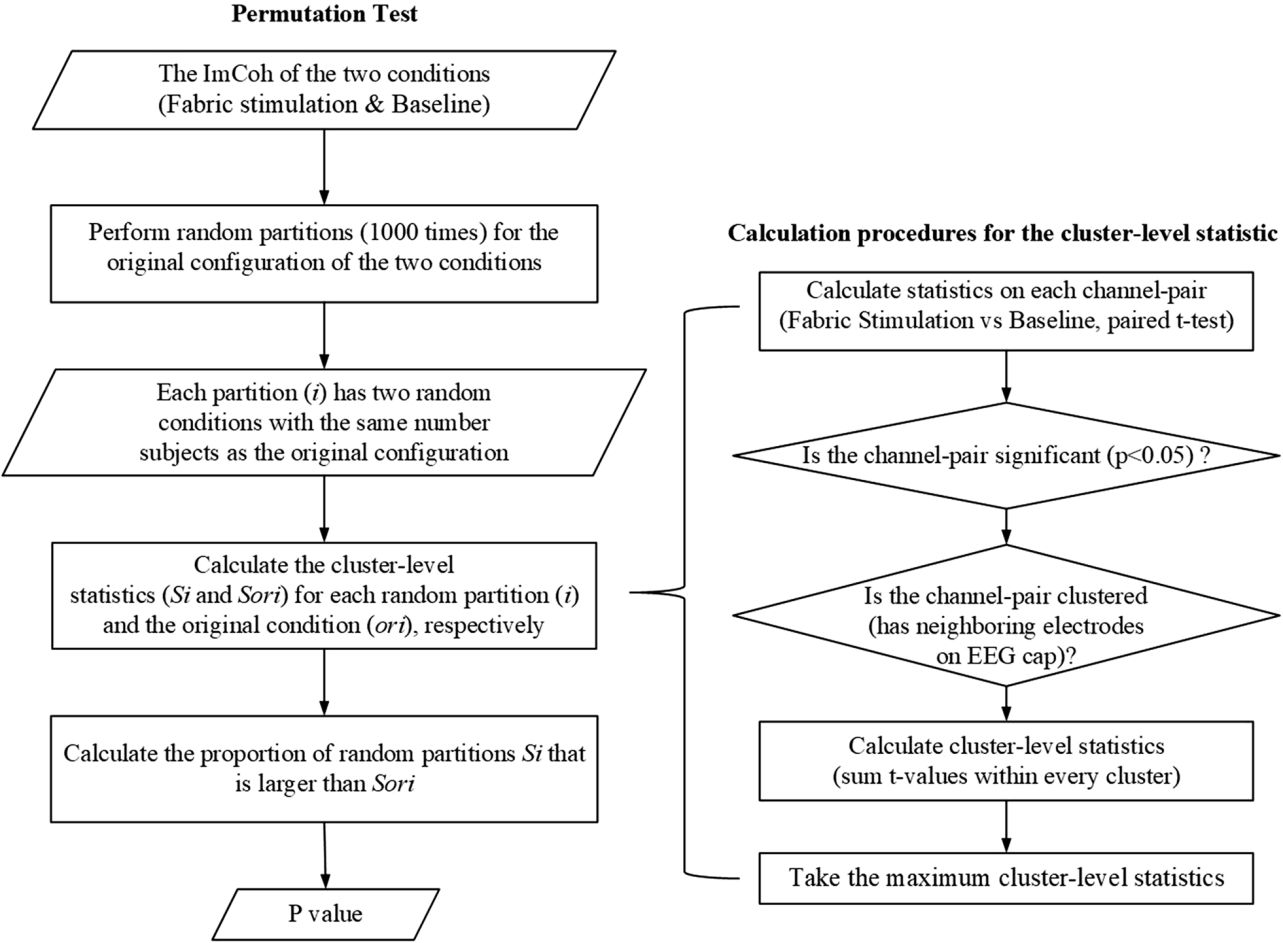
The flow chart on calculation procedures of the cluster-based permutation test

Based on the FC estimation of pair-wise ImCoh [24], the cluster-based permutation test [25] was used to obtain the fabric stimulation induced FC (SFC) representing the cortical “hot spot” in the stimulation. It was the illustration of locations of the most significant FC (Sig-FC) against the baseline with a significance level of 0.05 [25]. For each sub-band in beta and each subject group, the strength of FCs, i.e., the ImCoh values, of the fabric stimulation trials was statistically compared with that of the baseline trials across all subjects. Two main procedures were involved in the cluster-based permutation test, i.e., the calculation of cluster-level test statistic and the permutation test (Fig. 2). With the calculation of cluster-level statistics [37], it could retain Sig-FCs connected on adjacent EEG channels locally, i.e., the Sig-FC cluster, with maximum summation of statistics, i.e., cluster-level statistics, among all clusters. Meanwhile, it would reject the isolated Sig-FCs, i.e., without the Sig-FC on adjacent EEG channels, and those without the maximum cluster-level statistics [37]. In the subsequent analysis, the beta 2 band was chosen as the frequency band of interest, because only the beta 2 band had the SFC in the fabric stimulation to the two forearms in the unimpaired subjects after the cluster-based permutation test.

### Functional brain network at different scales

**Fig. 3:**
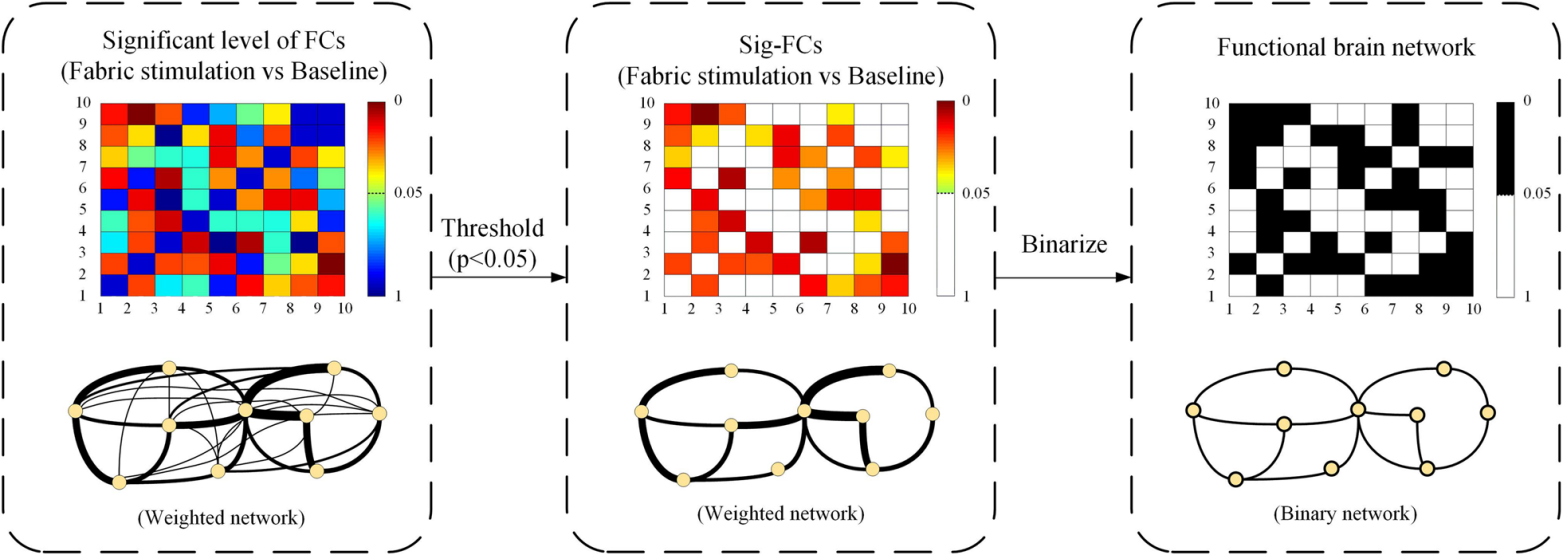
Estimation procedures of the function brain network based on the functional connectivity. A brain network with 10 EEG channels was used for illustration. The connectivity matrixes on the top panel were calculated from a statistical analysis between the FCs in the fabric stimulation and the baseline, in which the rows and columns represent EEG channels, matrix entries represent the FCs and the color scheme indicates the significant level of FCs. In the second and third connectivity matrix, a Sig-FC was represented as a colored entry and a black entry, respectively, while the FC without significance was represented as the white entry. The network graphs on the bottom panel were constructed from the corresponding connectivity matrix according to the graph theory, where nodes represent the EEG channels, links represent the FCs and the thickness of the links represents the significant level of FC

The overall connectivity pattern, representing the functional structure of the brain network in the stimulation, was described in terms of multiscale properties of the functional brain network, which was established based on all Sig-FCs, including the isolated Sig-FC and the ones with smaller statistical difference [38]. The functional structure of connectivity alteration in the brain in fine tactile sensation post-stroke was investigated both globally and locally by the multiscale topological analysis derived from graph theory, as previously applied in stroke motor functional studies [39]. Figure 3 shows the estimation procedures of the functional brain network with 10 EEG channels for illustration. Firstly, for each subject and the stimulation to each forearm, a statistical comparison between the FCs in the fabric stimulation trials and in the baseline trials (paired t-test corrected with false discovery rate (FDR), P < 0.05) was performed to maintain the significant FCs with respect to the baseline, as practiced previously in motor functional studies [39, 40]. These FCs were then transformed into binary values by using 1 and 0 to encode the Sig-FCs and the FCs without significance, respectively, thus generating an adjacency matrix representing the functional brain network (Fig. 3). In this work, the functional brain network was estimated based on the 62 EEG channels (the ground and reference were removed) on the whole brain.

Based on the estimated functional brain network, the topological indices were calculated at 3 scales as follows: (i) the entire brain (large scale), (ii) the 2 hemispheres (intermediate scale) and (iii) each EEG channel on the 2 hemispheres (small scale) [21], to illustrate the functional structure of the connectivity alteration in the brain during fine tactile sensation after stroke. These indices were first introduced by De Vico Fallani et al. to examine the functional neuroplasticity of impaired motor functions following the occurrence of a stroke [21].

#### Large-scale indices

In order to describe the efficient communication of the entire brain in response to the fine tactile stimulation, there were three large-scale indices, global efficiency (${E}_{glo}$), local efficiency (${E}_{loc}$) and smallworldness ($SW$) [21] to quantify the global properties of the functional brain network.

In graph theory, a functional brain network is mathematically represented as a graph matrix, in which EEG channels ($N=62$) were denoted by nodes and the Sig-FCs between EEG channel-pairs were denoted by links [21]. Given an adjacency matrix $A$ ($N\times N$) representing the functional brain network, if there is a link connecting the nodes $i$ and $j$, the corresponding entry in the matrix is ${a}_{ij}=1$, otherwise, ${a}_{ij}=0$. The global efficiency ${E}_{glo}$ is defined as the inverse of the averaged shortest path length between all pairs of nodes in the graph, which is calculated as follows:

2 $${E}_{glo}=\frac{1}{N(N-1)}{\sum }_{i\ne j\in A}\frac{1}{{d}_{ij}}$$

where ${d}_{ij}$ is the shortest path length among all the possible paths between nodes $i$ and $j$, represented by the minimum number of links on all possible paths between the two nodes. The ${E}_{glo}$ illustrated the efficiency of a functional brain network for integrating information from different brain regions in fine tactile sensation.

For each node $i$, its subgraph matrix ${A}_{i}$ consists of all its neighboring nodes. The local efficiency of the network is computed by averaging the local efficiency over all such subgraphs belonging to each node.

3 $${E}_{loc}=\frac{1}{N}{\sum }_{i\in A}E({A}_{i})$$

where $E\left({A}_{i}\right)$ is the local efficiency of the subgraph ${A}_{i}$ computed using the same equation as ${E}_{glo}$. The ${E}_{loc}$ demonstrated the brain network efficiency of processing information in partial regions (functional segregation).

The balance between functional integration and segregation is measured using the smallworldness, $SW$, which can be calculated as:

4 $$SW=\frac{{E}_{loc}/{E}_{loc}r}{{E}_{glo}/{E}_{glo}r}$$

where ${E}_{loc}r$ and ${E}_{glo}r$ are the efficiency values of the equivalent random graph with the same number of nodes and links as the brain network to be measured. The $SW>1$ implies that the network exhibits small-world properties, which is simultaneously segregated and integrated rather than a random network or a regular network [38][38]. Smallworldness is a snapshot of the ensemble network [42].

#### Intermediate-scale indices

The intermediate-scale indices measured the information interaction between and within the two hemispheres during the fabric stimulation, where two indices, interdensity ${K}_{inter}$ and intradensity ${K}_{intra}$, were used to examine the functional brain network at the hemispheric level by quantifying the density of links between and within the left (Lhemi) and right (Rhemi) hemispheres (i.e., the unaffected and affected hemispheres in the stroke group) [21].

Interdensity ${K}_{inter}$ is defined as the ratio of the actual number of links between the two hemispheres to all possible links between them. Given 2 predetermined sets of nodes, ${S}_{Lhemi}$ and ${S}_{Rhemi}$, corresponding to EEG channels of the left and right hemispheres, respectively, the ${K}_{inter}$ can be calculated as follows:

5 $${K}_{inter}=\frac{1}{{{N}_{s}}^{2}}{\sum }_{i\in {S}_{Lhemi},j\in {S}_{Rhemi}}A(i,j)$$

where $A$ ($N\times N$) is (the same adjacency matrix representing the functional network as mentioned in Eq. (1)) $A(i, j)$ is the value of the entry in the row $i$ and column $j$, ${N}_{s}$ is the total number of all possible nodes within each hemisphere. The interdensity ${K}_{inter}$ ranges from 0 to 1 and is proportional to the number of connected nodes between the two hemispheres. The increase of ${K}_{inter}$ was generally considered as the compensation from one hemisphere to the other hemisphere in the alteration of brain connectivity post-stroke [21].

Intradensity ${K}_{intra}$ is defined as the actual number of links in a hemisphere over the total number of all possible links in the same hemisphere, which is calculated as:

6 $${K}_{intra}(S)=\frac{2}{{{N}_{s}}^{2}-{N}_{s}}{\sum }_{i\ne j\in S}A(i,j)$$

where $S = S_{{Lhemi}} \left| {S_{{Rhemi}} } \right.$ is the left or the right hemisphere, $A(i, j)$ and ${N}_{s}$ have the same meaning as Eq. (5). The intradensity ${K}_{intra}$ ranges from 0 to 1 and is proportional to the number of connected nodes within one hemisphere. The difference between the ${K}_{intra}$ of the two hemispheres could reflect the hemispheric lateralization in the alteration of brain connectivity post-stroke [21].

#### Small-scale indices

Small-scale indices were applied to measure the involvement of local brain areas in response to the fabric stimulation. The local features of the functional brain network were extracted on each node (each EEG channel) using two indices, the interdegree ${D}_{inter}$ and the intradegree ${D}_{intra}$, which represent the respective centrality of a node with respect to the links between and within the two hemispheres [21].

The interdegree ${D}_{inter}$ is defined as the total number of links of a node from one hemisphere to those in the other hemisphere, which is calculated as:

7 $${D}_{inter}(i)={\sum }_{i\ne j\in {S}_{hemi}}A(i,j)B(i,j)$$

where ${{S}_{hemi}=S}_{Lhemi}\cup {S}_{Rhemi}$, $B\left(i,j\right)$ denotes whether $i$ and $j$ belong to the same hemisphere, $B\left(i,j\right)=1$ if $i$ and $j$ belong to different hemispheres, otherwise, $B\left(i,j\right)=0$. ${D}_{inter}$ ranges from $0$ to $N$, and could localize the main cortical area where the abnormal ${K}_{inter}$ occurs [21]. Changes in the ${D}_{inter}$ of particular nodes could implicate the affected contribution of the corresponding brain regions to information interaction with the contralateral hemisphere in the alteration of brain connectivity post-stroke [21].

The intradegree ${D}_{intra}$ is defined as the total number of links of a node to other nodes within the same hemisphere, which is calculated as:

8 $${D}_{intra}(i)={\sum }_{i\ne j\in S}A(i,j)$$

where $S = S_{{Lhemi}} \left| {S_{{Rhemi}} } \right.$ represents the left or the right hemisphere. The ${D}_{intra}$ ranges from $0$ to $N-1$, and could reflect the main cortical area where the abnormal ${K}_{intra}$ occurs. A node with high ${D}_{inter}$ or ${D}_{intra}$ is considered to be central, since its removal would cause a severe reduction of links. Changes in the ${D}_{intra}$ of specific nodes could implicate the contribution of the corresponding brain regions to the information interaction with other regions in the same hemisphere in the alteration of brain connectivity post-stroke [21].

Based on the calculated small-scale indices by Eq. (7) and Eq. (8) (i.e., interdegree ${D}_{inter}$ and intradegree ${D}_{intra}$), a statistical comparison was performed on the respective ${D}_{inter}$ and ${D}_{intra}$ values between the fabric stimulation to different forearms. This comparison could demonstrate the SFC asymmetry in local brain areas in the affected limb post-stroke by using the unaffected limb as a reference condition [6] [21]. Similar comparison was also applied to the unimpaired group by using the right limb (dominant) as a reference condition. After the confirmation of normal distribution by the Shapiro–Wilk test (P > 0.05) [43], the paired t-test (P < 0.05) was applied to conduct the statistical comparison between the fabric stimulation to the two forearms in each group. The false discovery rate (FDR) correction (P < 0.05) was then performed on all nodes for multiple comparisons correction [44]. Finally, the significant difference between the stimulation to the two forearms was visualized on each node by the topography of each group.

#### Statistical comparison for multiscale brain network indices

After the Shapiro–Wilk test of normality, data of each index, i.e., global efficiency (${E}_{glo}$), local efficiency (${E}_{loc}$), smallworldness ($SW$), interdensity ${K}_{inter}$ and intradensity ${K}_{intra}$, were confirmed to be normally distributed (P > 0.05). The paired t-test (P < 0.05) was used to compare the differences of each brain network index between the fabric stimulation to the affected limb (Alimb) and that to the unaffected limb (Ulimb) for the stroke group. The paired t-test was also applied to compare the brain network indices with respect to the stimulation to the left limb (Llimb) versus the right limb (Rlimb) for the control group. Statistical significance was set at 0.05 in this work. The level of statistical significance was also indicated at 0.01. All statistical analyses were performed using SPSS version 20 (SPSS, Chicago, IL).

## Results

### Alteration in SFC topology after stroke

**Fig. 4:**
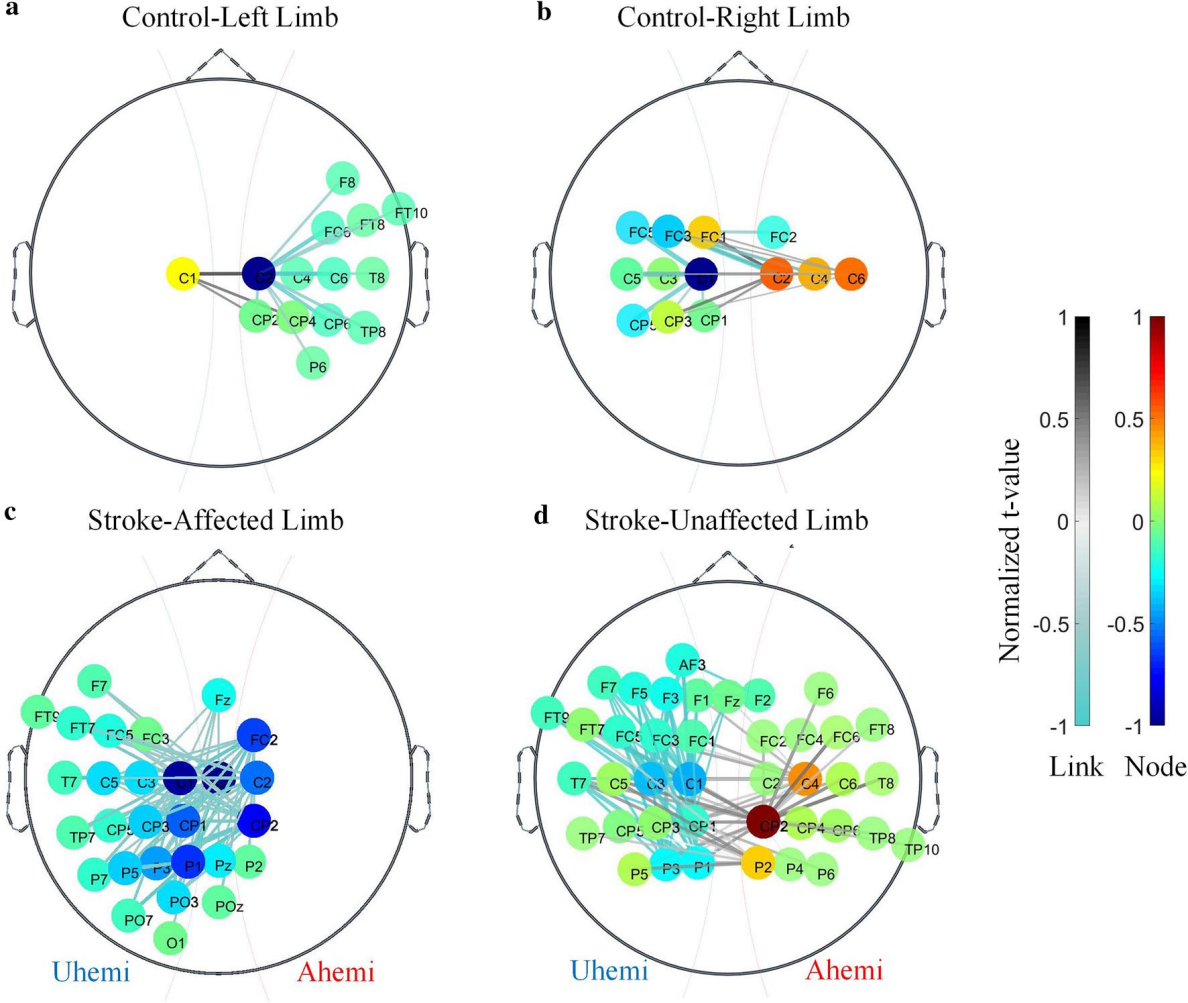
SFC topographies when the fabric stimulation applied to the right (**a**) and left (**b**) limbs of unimpaired subjects and when the stimulation applied to the affected (**c**) and the unaffected limbs (**d**) of stroke participants. Lines denote the SFC, calculated by a statistical comparison between the FCs in the fabric stimulation and in the baseline (P < 0.05, cluster-based permutation test). The color scheme and thickness of the lines represent the normalized t-value of the SFC. The colors of the nodes represent the sum of the normalized t-values on its connected SFC

Figure 4 shows the SFC topographies in response to the fabric stimulation to both forearms in the stroke and control groups. For the control group, the overall SFC tended to cover the sensorimotor area contralateral to the stimulated side with the most prominent change in the central cortical area of the S1 region. When the stimulation was applied to the left forearm (Fig. 4a), the distribution of SFC was observed to have a hemispheric lateralization (HL), which showed wider distribution over the contralateral right hemisphere than the ipsilateral hemisphere. The intensity of SFC was decreased in the contralateral side (i.e., contralateral decrease) but increased in the ipsilateral side (i.e., ipsilateral increase). In terms of the distribution, the SFC showed two clusters of connections centered on the central cortical area (C1 and C2, respectively). It mainly covered the S1 (C4, C6, CP2, CP4, CP6, FC6) and partly covered the temporal (FT8, FT10, T8, TP8), frontal (F8) and parietal lobes (P6). The hemispheric lateralization degree was 92% (i.e., 13 contralateral channels / 14 total number of channels on the SFC topography). In terms of the intensity, the mean intensity was–29 for the SFCs of contralateral hemisphere and + 8.88 for those of the ipsilateral.

When stimulating the right forearm in the control group (Fig. 4b), a specular behavior was observed in the SFC pattern, i.e., hemispheric lateralization, in which the SFCs had a wider distribution over the contralateral hemisphere than the ipsilateral, as in the stimulation conducted on the left forearm. Another SFC pattern similar to the left limb stimulation was that the mean intensity of the SFC in the two hemispheres also showed an ipsilateral increase and a contralateral decrease in terms of connection strength. Specifically, 5 clusters of SFCs were mainly centered on the central cortical area (C1, C2, C6, FC3) and distributed mainly over the S1 area (C3, C4, C5, FC1, FC2, FC5, CP1, CP3) and partly over the central-parietal areas (CP5). The hemispheric lateralization degree was 69% (i.e., 9 contralateral channels / 13 total number of channels on the SFC topography). The mean intensity for the SFCs of contralateral channels was – 1.84, while the mean intensity for SFCs of ipsilateral channels was + 21.64.

In contrast to the controls, the stroke group exhibited a more intermingled structure of the SFC topography in the fabric stimulation. When the stimulation was applied to the affected forearm in the stroke group (Fig. 4c), the SFC did not show the typical hemispheric lateralization as in the control, but rather more involved brain regions and altered mean intensity in the two hemispheres. In terms of the distribution, the SFC had 9 clusters of connections covering most of the somatosensory (C1-C3, Cz, C5, FC2, FC3, FC5, CP1-CP3, CP5, PO3, P1-P3, Pz) and part of the frontal (Fz, F7, FT7, FT9), temporal (T7, TP7), parietal (P5, P7, PO7, POz) and occipital (O1) areas. The hemispheric lateralization degree was 14% (i.e., 4 contralateral channels / 28 total number of channels on the SFC topography). In terms of the intensity, the mean intensity for the SFCs of contralateral channels and ipsilateral channels was − 14.46 and − 211.55, respectively.

When stimulating the unaffected limb of the stroke group (Fig. 4d), the distribution of the SFC had a weaker hemispheric lateralization and more involved brain regions than those of the control group. However, the mean intensity of the SFC in the two hemispheres was similar to the control group which also showed an ipsilateral increase and a contralateral decrease. Specifically, the SFC had 10 clusters of connections not only over the sensorimotor area (C1-C6, FC1-FC6, CP1-CP6, P1-P4), but also over the frontal (F1-F3, F5-F7, Fz, AF3, FT7-FT9), temporal (T7, T8, TP7, TP8, TP10), and parietal (P5, P6) areas, which was wider than that of the control group. The hemispheric lateralization degree was 53% (i.e., 21 contralateral channels / 40 total number of channels on the SFC topography). In terms of the intensity, the mean intensity for the SFCs of contralateral channels and ipsilateral channels was − 52.35 and + 54.20, respectively.

### Multiscale brain network properties after stroke

#### Large-scale properties

**Fig. 5:**
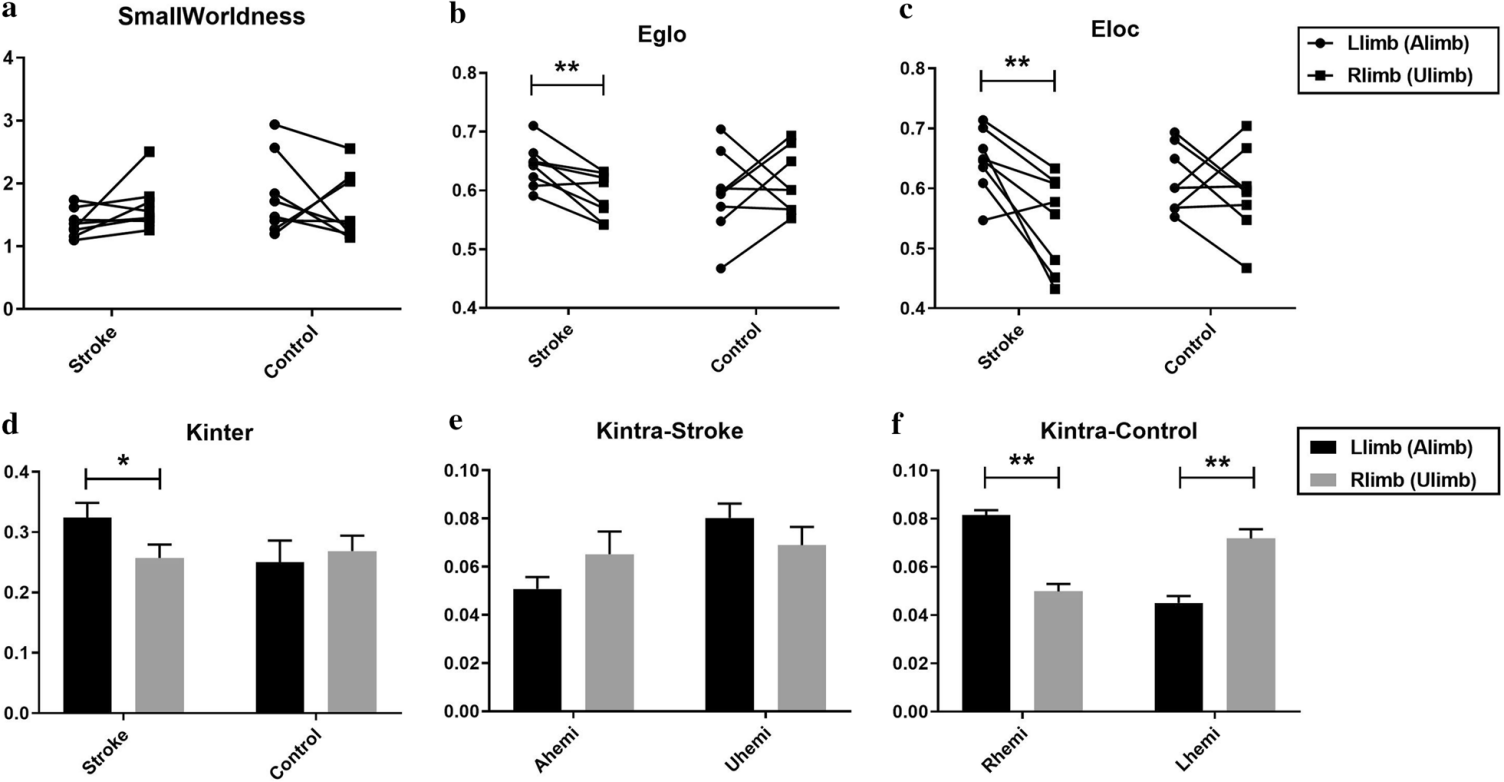
Large-scale and intermediate-scale brain network properties for the stroke and control groups. **a**–**c** The smallworldness $SW$, global-efficiency ${E}_{glo}$ and local-efficiency ${E}_{loc}$ in the affected limb stimulation (left limb for the controls, round dots) and unaffected limb stimulation (right limb for the controls, square dots) as linked by line for each subject in both groups. **d** Mean values of the interdensity ${K}_{inter}$ in the affected limb (left limb, black bars) and unaffected limb (right limb, gray bars) stimulation for the two groups. **e**, **f** are the intradensity ${K}_{intra}$ of the unaffected (right) hemisphere and affected (left) hemisphere for the stroke and control groups, respectively. The vertical line indicates the Standard Error of Mean (SEM). The asterisk denotes the significant (* denotes P < 0.05 and ** denotes P < 0.01, paired t-test) differences with regard to the stimulation to the two forearms

The large-scale functional brain network was analyzed by comparing the $SW$, ${E}_{glo}$ and ${E}_{loc}$ indices between the stimulation to different limbs (Fig. 5a, b and c; Table 3). For the stroke group, the brain networks in stimulation to both limbs showed small-world properties (i.e., $SW>1$), while no significant difference was found with regard to the $SW$ when stimulating different limbs (P > 0.05, paired t-test, Table 3; Fig. 5a, left panel). Additionally, the global efficacy ${E}_{glo}$ (Fig. 5b, left panel) and the local efficacy ${E}_{loc}$ (Fig. 5c, left panel) were significantly higher in the stimulation to the affected limb than the unaffected limb (${E}_{glo}$: P = 0.006, effect size (EF) = 0.563; ${E}_{loc}$: P = 0.009, EF = 0.563, paired t-test, Table 3). For the unimpaired group, the brain networks in stimulation to different limbs also exhibited small-world properties. However, there was no significant difference with regard to any large-scale indices (i.e., $SW$, ${E}_{glo}$ and ${E}_{loc}$) in stimulation to the two limbs in the unimpaired (P > 0.05, paired t-test, Table 3; Fig. 5a, b and c, right panel).

**Table 3:** The means and 95% confidence intervals of large-scale and intermediate-scale brain network indices with paired t-test probabilities in the fabric stimulation to the two forearms in the stroke and control groups (* denotes P < 0.05 and ** denotes P < 0.01)

Brain network Index	Group	Llimb (Alimb)	Rlimb (Ulimb)	Paired t-test
		Mean (95% confidential interval)		P-value (effect size)
Smallworldness	Stroke	1.37 (1.18–1.55)	1.64 (1.32–1.97)	0.116 (0.250)
	Control	1.80 (1.27–2.33)	1.62 (1.18–2.07)	0.511(0.188)
${E}_{glo}$	Stroke	0.64 (0.61–0.67)	0.59 (0.56–0.62)	0.006** (0.563)
	Control	0.59 (0.53–0.65)	0.61 (0.57–0.66)	0.522 (0.130)
${E}_{loc}$	Stroke	0.65 (0.60–0.69)	0.54 (0.48–0.61)	0.009** (0.563)
	Control	0.61 (0.57–0.66)	0.59 (0.53–0.65)	0.522 (0.126)
${K}_{inter}$	Stroke	0.32 (0.26–0.38)	0.26 (0.20–0.31)	0.038* (0.375)
	Control	0.25 (0.16–0.34)	0.27 (0.21–0.33)	0.700 (0.125)
${K}_{intra}$- Rhemi (Ahemi)	Stroke	0.05 (0.04–0.06)	0.07 (0.04–0.09)	0.280 (0.125)
	Control	0.08 (0.07–0.09)	0.06 (0.05–0.06)	0.002** (0.625)
${K}_{intra}$- Lhemi (Uhemi)	Stroke	0.08 (0.07–0.09)	0.07 (0.05–0.09)	0.316 (0.063)
	Control	0.05 (0.04–0.05)	0.07 (0.06–0.08)	0.006** (0.813)

#### Intermediate-scale properties

At the intermediate topological scale, the connectivity density of inter- and intra-hemispheric connectivity was compared in the stimulation to the two forearms. For the stroke group, the interdensity ${K}_{inter}$ was significantly higher (P = 0.038, EF = 0.375, paired t-test, Table 3; Fig. 5d, left panel) in the affected limb stimulation with respect to the unaffected limb stimulation. The intradensity ${K}_{intra}$ within the affected hemisphere did not differ between the stimulation to the affected and unaffected limbs (P > 0.05, paired t-test, Table 3; Fig. 5e, left panel). Similarly, no significant difference was observed for the ${K}_{intra}$ within the unaffected hemisphere between the stimulation to the two limbs (P > 0.05, paired t-test, Table 3; Fig. 5e, right panel). For the unimpaired group, no significant difference was observed for the interdensity ${K}_{inter}$ (P > 0.05, paired t-test, Table 3; Fig. 5d, right panel). However, a significantly higher ${K}_{intra}$ was identified on the hemisphere contralateral to the stimulation side (Rhemi / Lhemi) in the fabric stimulation to the Llimb (or Rlimb) than the Rlimb (or Llimb) (Rhemi: P = 0.002, EF = 0.625; Lhemi: P = 0.006, EF = 0.813; paired t-test, Table 3; Fig. 5f).

#### Small-scale properties

**Fig. 6:**
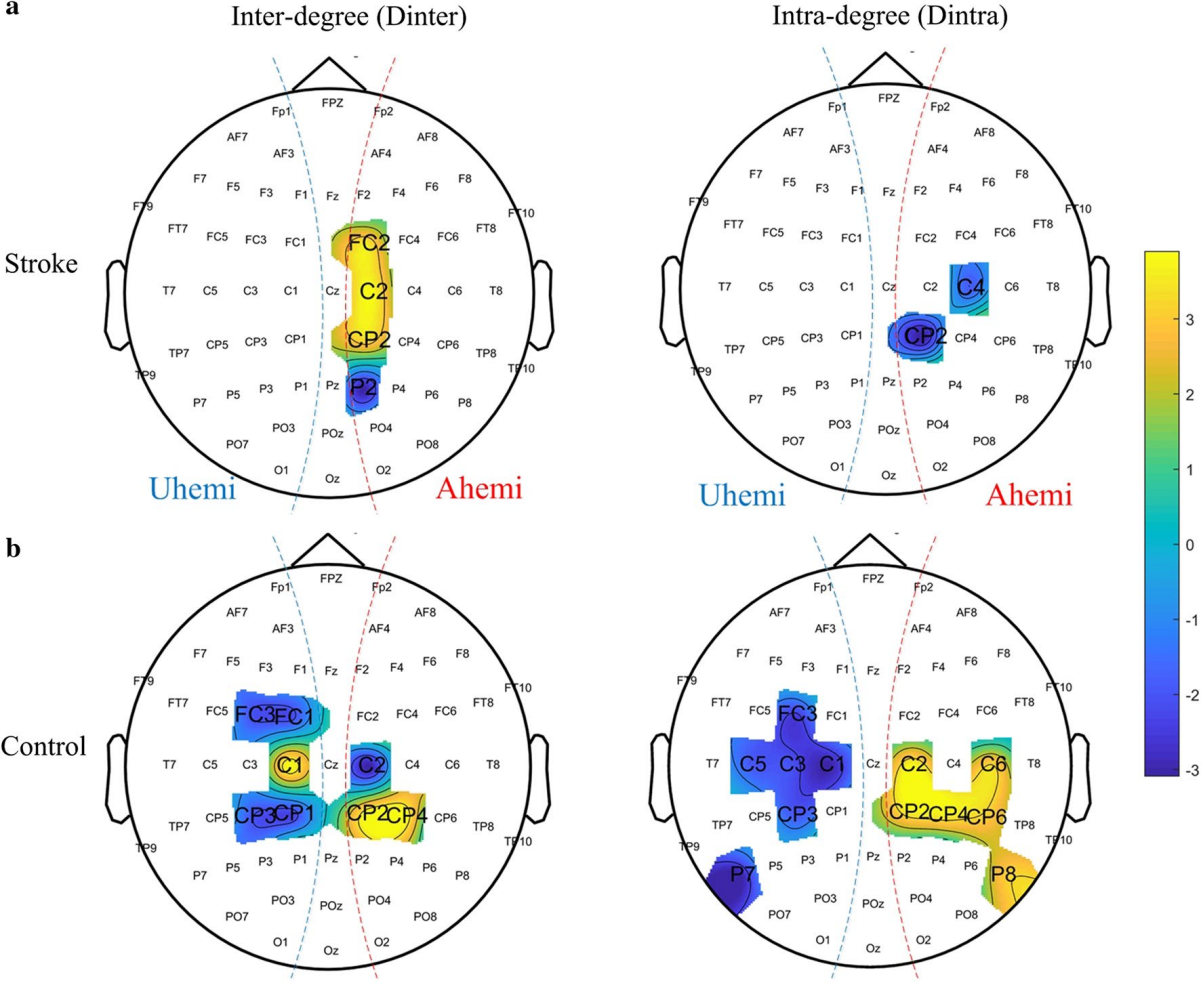
Topographies of the statistical significance related to the small-scale brain network properties for stroke (**a**) and control groups (**b**). Only the significant channels (P < 0.05, Paired t-test with FDR correction P < 0.05) in the affected limb stimulation versus the unaffected limb stimulation (Llimb-versus-Rlimb stimulation for the control group) are shown on the topography. The inter-degree ${D}_{inter}$ and intra-degree ${D}_{intra}$ are represented on the left panel and the right panel, respectively. The color scheme denotes the level of statistical difference (t-value) of each channel. The yellow and blue colors represent the significant higher and significant lower small-scale values in the Alimb than the Ulimb stimulation, respectively

For the stroke group, the interdegree, ${D}_{inter}$, (Fig. 6a, left panel) showed significant differences only in the somatosensory area of the affected hemisphere (P < 0.05, paired t-test with FDR correction). It was significantly higher for channels FC2, C2 and CP2 in the S1 area but significantly lower for channel P2 in the secondary somatosensory area (S2) in the stimulation to the affected limb than the unaffected limb (0.29 < EF < 0.50). Additionally, when the stroke affected limb was stimulated, the intradegree, ${D}_{intra}$, (Fig. 6a, right panel) showed a significant difference only in the S1 area of the contralateral affected hemisphere (C4 and CP2), compared with the unaffected limb stimulation (C4: EF = 0.36; C6: EF = 0.29).

For the control group (Fig. 6b), the brain regions with significantly different interdegree or intradegree between the stimulation to different forearms tended to distribute symmetrically over the two hemispheres (P < 0.05, paired t-test with FDR correction). Specifically, the interdegree, ${D}_{inter}$, (Fig. 6b, left panel) was significantly higher for channels C1, CP2 and CP4, but significantly lower for the opposite channels C2, CP1, CP3, FC1 and FC3 in the left limb stimulation versus the right limb stimulation (0.21 < EF < 0.29). Additionally, compared with stimulation to the right forearm, stimulation to the left limb elicited significantly higher intradegree ${D}_{intra}$ (Fig. 6b, right panel) in the somatosensory (C2, C6, CP2, CP4 and CP6) and parietal regions (P8) of the contralateral right hemisphere, but significantly lower in the opposite somatosensory region (C1, C3, C5, CP3, FC3 and P7) (0.14 < EF < 0.36).

## Discussion

In this work, we investigated the EEG-derived FC and the functional brain networks at 3 scales, i.e., the large, intermediate and small scales, in response to the fine tactile stimulation via textile fabrics to persons with chronic stroke and unimpaired controls. The results demonstrated that the post-stroke alteration of cortical connectivity in response to fine tactile stimulation could be characterized by altered hemispheric lateralization, additionally involved brain regions, increased interhemispheric connectivity and increased global and local efficiencies in the functional brain network.

### Alterations of hemispheric lateralization after stroke

An altered hemispheric lateralization was noted in the SFC topological distribution in response to the fabric stimulation to the affected forearm (HL degree, 14%) in the stroke group, compared with the unaffected forearm (HL degree, 53%) and either forearms of the control group (HL degrees, 92% and 69% for the Llimb and Rlimb stimulation, respectively) (Fig. 4). This altered hemispheric lateralization in SFC distribution was concomitant with the alterations in the SFC intensity after stroke: The SFC intensity decreased in both hemispheres when stimulating the affected limb in the stroke, in contrast to the patterns for the control and when stimulating the unaffected limb of the stroke (i.e., the contralateral decrease and ipsilateral increase). These findings indicated a redistributed pattern from the contralateral hemisphere to the ipsilateral hemisphere in the SFC topology in response to the fabric stimulation to the stroke affected forearm. This could be attributed to the compensatory effect from the “intact” contralesional hemisphere in the process of neural plasticity and regeneration, as previously suggested in motor functional studies [45]. The damage to the brain neuron axons following the occurrence of a stroke would trigger the remaining axons near denervated regions to sprout new collaterals and to form synapses with denervated neurons [46]. This neuron regeneration process proceeds in different brain regions that previously had anatomical connections with the injured region [47]. Many previous studies confirmed that the contralesional cortex plays an important role in compensatory movements related to motor impairments and being a dominant force in shaping post-stroke neuroplasticity [45]. The results in this work suggested that similar compensatory effects from the contralesional hemisphere could also occur after the sensory impairment post-stroke. The SFC analysis could provide a potential evaluation method on the tactile impairments in the sensorimotor rehabilitation.

The SFC topographies (Fig. 4) of the control and stroke groups demonstrated more bilateral involvement of the hemispheres during the stimulation to the dominate limb, or unaffected limb, than the stimulation to the non-dominated limb, or the affected limb. Right-handed participants were recruited in the control group, whose right hemisphere was found to be more involved in tactile stimulation to both upper limbs than the left. Similar findings related to more bilateral hemisphere involvements during sensory stimulation to the dominant upper limb have also been reported in fMRI studies on unimpaired subjects [48–50]. The more intensive bilateral involvement of the hemispheres during the stimulation to the unaffected upper limb of the chronic stroke could be related to the compensatory effects after long-term disability, where the unaffected limb was habitually restored as the dominant limb in the daily life [28].

In the functional structure of the connectivity alteration in the brain, the redistributed SFC pattern (i.e., altered hemispheric lateralization) post-stroke was verified by the intradensity ${K}_{intra}$ (Fig. 5e, f) and further localized by the intradegree ${\mathrm{D}}_{\mathrm{intra}}$ (Fig. 6, right panel). For the unimpaired subjects, the hemispheric lateralization was indicated by the significantly greater contribution of either hemispheres to the contralateral limb stimulation than the ipsilateral limb (Fig. 5f), which was mainly located in the somatosensory region (Fig. 6b, right panel). This suggested that there was an SFC symmetry in the somatosensory region in the fine tactile stimulation to either limbs in the unimpaired persons. Previous findings based on fMRI indicated that different sensory stimuli (vibrotactile, pressure, warmth, and coolness) on the right hand or electrical stimulation of the right median nerve produced increased probability of activation in the S1 region contralateral to the stimulated side and a higher probability of deactivation in ipsilateral S1 [50–52]. In this study, similar findings observed in fMRI were also obtained in the EEG patterns during the transient sensory stimulation for the control group.

For the stroke group, although no significant difference was observed in the hemispheric level (Fig. 5e), the altered hemispheric lateralization was characterized by significantly deceased intra-hemispheric connectivity in the ipsilesional S1 region in the stimulation to the affected forearm, compared to the stimulation to the unaffected forearm (Fig. 6a, right panel). These results possibly suggested that the asymmetric SFC pattern was related to the transient deactivation of the ipsilesional S1 region in the reorganized brain structure post-stroke. Although the stroke participants in a resting state exhibited an increased intra-hemispheric FC in ipsi-lesional S1 measured by fMRI compared with the unimpaired persons [9], the EEG patterns in this work indicated the deactivation of the ipsilesional S1 region in response to the transient sensory stimulation post-stroke. Additionally, in the unimpaired subjects, the increased activation of the contralateral S1 region in the symmetrical activation pattern was observed to be a distinct cortical response to tactile stimulation when brushing the unilateral hand skin with a sponge [50]. Our findings in persons with chronic stroke highlighted that the transient fine tactile stimulation to different limbs produced an asymmetric connectivity pattern along with the deactivation of the ipsilesional S1 region in the altered brain connectivity. By means of the EEG-derived FC, this work provided insights into the alteration of neural responses in transient fine tactile sensation after stroke.

### Wider topological distribution of SFC after stroke

Involuntary attention is an exogenous attention driven by transient stimuli compared to the endogenous voluntary attention which is directed by goals [53]. In the experimental paradigm of this study, voluntary attention was less likely to occur because of the minimized cognitive processes, during which the participant was asked to avoid active mental tasks and wore the eye mask and ear plugs to minimize the visual and audio interferences. Meanwhile, the involuntary attention could be recruited by the fabric stimulation as the only exogenous attentional input to the participant. For the control group, the topological distribution of SFC was observed to be primarily over the somatosensory region when the fine tactile stimulation was applied to either forearms (Fig. 4a, b). In contrast, additional brain regions, including the frontal, temporal and parieto-occipital areas, were noted in the wider topological distribution of SFC when the fine tactile stimulation was applied to either forearms of the stroke group (Fig. 4c, d), particularly for the affected forearm. These areas were related to parts of the distributed attention networks [54]. It suggested that more neural network resources related to involuntary attention were attracted in the fine tactile sensation in the stroke group than the control, possibly due to a compensatory effect after the tactile impairments post-stroke, as previously observed in motor impairments post-stroke [55].

As with the compensation from the contralesional hemisphere observed in this work, the compensation from the attention networks could be recruited by the neural regenerative process post-stroke [47]. Specifically, the degeneration of neurons after a stroke led to widespread regenerative counter-reactions, which would elicit a reestablishment of the synaptic connectivity patterns across surviving neurons, particularly in tissue and areas near the core region of the damage [46]. As adjacent regions of the somatosensory region, the distributed attention networks could potentially undergo the regenerative neuron process and exhibit reestablished synaptic connectivity patterns post-stroke [56]. Additionally, since focal attention is essential to any sensation or learning tasks [53], the findings in this work reflected that the recruitment of higher-level attention functions, i.e., involuntary attention, potentially supplemented to the lower-level tactile perceptual functions in persons with chronic stroke. The previous rsfMRI studies suggested the importance of resting-state FC between attention networks and the somatosensory region in improved touch sensation post-stroke, which was represented by a positive correlation between the outcomes of the tactile discrimination test and the number of FC in the attention networks [8, 57]. However, the neural activities in the attention network induced by transient tactile stimulation have not been directly detected. In this study, the additionally involved attention networks in the SFC topological pattern uncovered the compensatory contribution from distributed attention networks to the neural activities in transient tactile sensation at the chronic stage post-stroke.

### Increased interhemispheric FC after stroke

For the number of interhemispheric connectivity, there was also no significant difference at the hemispheric level in the control group (Fig. 5d). However, significant differences at regional level were observed in the somatosensory region when stimulating different forearms in the control group (Fig. 6b, left panel). In contrast, significantly increased number of interhemispheric connectivity at the hemispheric level was observed when stimulating the affected limb, compared to the stimulation to the unaffected limb in the stroke group (Fig. 5d). This hemispheric-level change was mainly located in the ipsilesional somatosensory region in the stroke participants (Fig. 6a, left panel). Two main reasons could contribute to the significant increase of interhemispheric connectivity in the affected limb stimulation than the unaffected limb, i.e., the altered interhemispheric inhibition [58] and the enhanced static interhemispheric connectivity in chronic stroke [59]. Firstly, similar findings on the enhanced interhemispheric connectivity have been reported in previous fMRI studies on motor tasks post-stroke, showing additional interhemispheric inhibition from contralesional to ipsilesional M1[58, 60]. As the recruited stroke subjects were at a very chronic stage, the observation in this work could also be related to the re-establishment of static interhemispheric connectivity, which was found to be restored in the sub-acute phase and enhanced to a level higher than the unimpaired controls in the chronic phase post-stroke [7, 59]. The finding in this work also suggested the contribution of interhemispheric connectivity in the ipsilesional somatosensory region to the alteration of brain connectivity in fine tactile sensation after stroke. This could be associated with the compensation from the contralesional hemisphere to the ipsilesional hemisphere and could potentially lead to the altered hemispheric lateralization observed in the SFC distribution, given the important role of interhemispheric connectivity in interhemispheric mutual control [61]. For unimpaired subjects, previous studies on unimanual movements indicated that the interhemispheric connectivity could facilitate the hemispheric lateralization by inhibiting ipsilateral hemisphere and transferring lateralized information via corpus callosum [62]. It was also found that the corpus callosum was responsible for the interhemispheric information transfer in tactile discrimination, as indicated in a study on primates [63]. Additionally, the resting-state interhemispheric connectivity in the sensorimotor system was reported to have a compensatory neural plasticity in the motor recovery [7, 59, 61]. Our findings suggested that the compensation represented by the interhemispheric connectivity in ipsilesional somatosensory region also occurred in the neural processes of transient fine tactile sensation post-stroke, possibly implemented through the transcallosal pathway. According to the principle of neural regeneration after a focal lesion [46], the changed interhemispheric connectivity in ipsilesional somatosensory region observed in this work might indicate the sprouting out of axons in the transcallosal pathway to establish new connections and projection patterns from the contralesional hemisphere to the ipsilesional hemisphere in the neural circuits related to the fine tactile sensation in persons with chronic stroke.

### Increased cortical activity after stroke

In terms of the overall performance of the brain network, no significant difference was found for the smallworldness ($\mathrm{SW}>1$) between the stimulation to the two forearms in both subject groups (Fig. 5a). This possibly suggested that the brain networks in response to fine tactile stimulation post-stroke still contained the networks that are simultaneously segregated and integrated, as in the control group, rather than a random network or a regular network [41, 42]. In terms of the global and local efficiencies of the brain network, for the control group, no significant difference was found with regard to either ${E}_{glo}$ or ${E}_{loc}$ when stimulating the different forearms (Fig. 5b, c). However, for the stroke group, the fabric stimulation to the affected forearm was characterized by the significant increase of ${E}_{glo}$ and ${E}_{loc}$ than the stimulation to the unaffected forearm (Fig. 5b, c). This suggested that there was a higher tendency of the brain network to form groups of regions with tight inter- and intra- connections in neural circuits related to fine tactile sensation by the affected limb after stroke, as suggested in [21]. In the context of networks, the presence of highly connected non-overlapping groups implies that the network divided naturally into sets of nodes with dense connections internally and externally between sets [41]. The increase of ${E}_{glo}$ and ${E}_{loc}$ observed in this study could be attributed to the outgrowth of new connections in additionally involved attention regions and the compensation from the contralesional hemisphere in fine tactile sensation after stroke. A previous study showed that the involvement of additional brain regions could improve the cortical efficacy, suggesting that a more efficient but less economical configuration was adopted by the brain networks in the increased cortical activity as in the working memory task [64]. Additionally, given the fact that higher global and local efficiencies were generally observed in unimpaired subjects than the stroke persons [21], one might expect the reorganized brain post-stroke to possess reduced global and local efficiencies, as the brain deviated from “normal function”. However, a previous study indicated that higher global and local efficiencies were positively correlated with the restoration of motor function post-stroke [23]. In other words, no consensus was reached regarding the changes of global and local efficiency in relation to the functional deficits post-stroke, particularly in the motor deficits [23]. Our findings of the global and local efficiency analysis could indicate the increased cortical activity in fine tactile sensation in persons with chronic stroke, possibly due to the additionally involved attention regions related to involuntary attention and the compensation from the contralesional hemisphere.

### Limitation

The EEG and fMRI will be synchronously recorded during the fine tactile sensation for a more comprehensive understanding on the sensory impairment post-stroke in our future work, due to the limitations of using EEG as the only measure compared to fMRI, e.g., lower spatial resolution, ill-posed inverse problem in estimation of activation maps and limitation of the response detection at the cortical level.

## Conclusions

In this study, we investigated the post-stroke alteration of cortical connectivity in response to fine tactile stimulation via the textile fabrics by EEG-derived functional connectivity analysis in persons with chronic stroke. The results demonstrated that the alteration of cortical connectivity in fine tactile sensation post-stroke could be characterized by altered hemispheric lateralization, additionally involved brain regions, increased interhemispheric FC and increased global and local efficiencies in the functional brain network. Specifically, compensatory effects from the contralesional hemisphere and the distributed attention networks related to involuntary attention were visualized by the SFC topology when stimulating the affected forearm in the stroke group, compared with those of the controls and when stimulating the unaffected forearm. The compensation from the contralateral hemisphere could be implemented by the interhemispheric connectivity from the ipsilesional somatosensory region via the transcallosal pathway in neural circuits related to fine tactile sensation. Stroke participants also exerted increased cortical activities represented by the increased global and local efficacies in the large-scale brain network in fine tactile stimulation, possibly due to the compensatory effects from the additionally involved attention regions related to involuntary attention and the contralesional hemisphere.

## Data Availability

The datasets analyzed in the current study are not publicly available, because it has been stated in the consent approved by the Human Subjects Ethics Sub-Committee of the Hong Kong Polytechnic University that the results of the experiment may be published, but the individual results should be kept confidentially for each subject.

## Abbreviations

EEG: Electroencephalogram
FC: Functional connectivity
SFC: Fabric stimulation induced functional connectivity
HL: Hemispheric lateralization
rsfMRI: Resting-state functional magnetic resonance imaging
S1: Primary somatosensory cortex
SMA: Supplementary motor area
S2: Secondary somatosensory area
FMUE: Fugl-Meyer Assessment
MMSE: Mini-Mental State Examination
FMA: Fugl-Meyer Assessment
FIM: Functional Independence Measurement
MAS: Modified Ashworth Score
ARAT: Action Research Arm Test
ImCoh: Imaginary coherence
Sig-FC: Significant FC
FDR: False discovery rate
*E*_glo_: Global efficiency
*E*_loc_: Local efficiency
*SW*: Smallworldness
Llimb: Left limb
Rlimb: Right limb
Alimb: Affected limb
Ulimb: Unaffected limb
Lhemi: Left hemisphere
Rhemi: Right hemisphere
Ahemi: Affected hemisphere
Uhemi: Unaffected hemisphere
*K*_inter_: Interdensity
*K*_intra_: Intradensity
*D*_inter_: Interdegree
*D*_intra_: Intradegree
EF: Effect size
